# User Experience of Persons Using Ingestible Sensor–Enabled Pre-Exposure Prophylaxis to Prevent HIV Infection: Cross-Sectional Survey Study

**DOI:** 10.2196/53596

**Published:** 2024-05-03

**Authors:** Sara Browne, Anya Umlauf, David J Moore, Constance A Benson, Florin Vaida

**Affiliations:** 1 Division of Infectious Diseases and Global Public Health, University of California San Diego, San Diego, CA, United States; 2 Specialists in Global Health, Encinitas, CA, United States; 3 Department of Psychiatry, University of California San Diego, San Diego, CA, United States; 4 Department of Family Medicine and Public Health, University of California San Diego, San Diego, CA, United States

**Keywords:** ingestible sensor, sensor, sensors, oral, UX, user experience, HIV prevention, medication adherence, HIV, prevention, prophylaxis, STI, STD, sexually transmitted, sexual transmission, drug, drugs, pharmacy, pharmacies, pharmacology, pharmacotherapy, pharmaceutic, pharmaceutics, pharmaceuticals, pharmaceutical, medication, medications, adherence, compliance, sexually transmitted infection, sexually transmitted disease

## Abstract

**Background:**

A digital health technology’s success or failure depends on how it is received by users.

**Objectives:**

We conducted a user experience (UX) evaluation among persons who used the Food and Drug Administration–approved Digital Health Feedback System incorporating ingestible sensors (ISs) to capture medication adherence, after they were prescribed oral pre-exposure prophylaxis (PrEP) to prevent HIV infection. We performed an association analysis with baseline participant characteristics, to see if “personas” associated with positive or negative UX emerged.

**Methods:**

UX data were collected upon exit from a prospective intervention study of adults who were HIV negative, prescribed oral PrEP, and used the Digital Health Feedback System with IS-enabled tenofovir disoproxil fumarate plus emtricitabine (IS-Truvada). Baseline demographics; urine toxicology; and self-report questionnaires evaluating sleep (Pittsburgh Sleep Quality Index), self-efficacy, habitual self-control, HIV risk perception (Perceived Risk of HIV Scale 8-item), and depressive symptoms (Patient Health Questionnaire–8) were collected. Participants with ≥28 days in the study completed a Likert-scale UX questionnaire of 27 questions grouped into 4 domain categories: overall experience, ease of use, intention of future use, and perceived utility. Means and IQRs were computed for participant total and domain subscores, and linear regressions modeled baseline participant characteristics associated with UX responses. Demographic characteristics of responders versus nonresponders were compared using the Fisher exact and Wilcoxon rank-sum tests.

**Results:**

Overall, 71 participants were enrolled (age: mean 37.6, range 18-69 years; n=64, 90% male; n=55, 77% White; n=24, 34% Hispanic; n=68, 96% housed; and n=53, 75% employed). No demographic differences were observed in the 63 participants who used the intervention for ≥28 days. Participants who completed the questionnaire were more likely to be housed (52/53, 98% vs 8/10, 80%; *P*=.06) and less likely to have a positive urine toxicology (18/51, 35% vs 7/10, 70%; *P*=.08), particularly methamphetamine (4/51, 8% vs 4/10, 40%; *P*=.02), than noncompleters. Based on IQR values, ≥75% of participants had a favorable UX based on the total score (median 3.78, IQR 3.17-4.20), overall experience (median 4.00, IQR 3.50-4.50), ease of use (median 3.72, IQR 3.33-4.22), and perceived utility (median 3.72, IQR 3.22-4.25), and ≥50% had favorable intention of future use (median 3.80, IQR 2.80-4.40). Following multipredictor modeling, self-efficacy was significantly associated with the total score (0.822, 95% CI 0.405-1.240; *P*<.001) and all subscores (all *P*<.05). Persons with more depressive symptoms reported better perceived utility (*P*=.01). Poor sleep was associated with a worse overall experience (−0.07, 95% CI −0.133 to −0.006; *P*=.03).

**Conclusions:**

The UX among persons using IS-enabled PrEP (IS-Truvada) to prevent HIV infection was positive. Association analysis of baseline participant characteristics linked higher self-efficacy with positive UX, more depressive symptoms with higher perceived utility, and poor sleep with negative UX.

## Introduction

**Figure 1.**
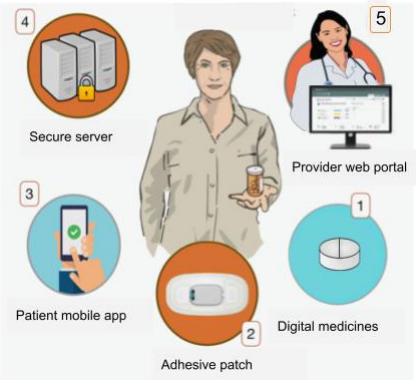
Depiction of the components of the Digital Health Feedback System (DHFS). (1) At home, the patient takes the digitized medicine. The ingestible sensor activates in the stomach, and its serial number is captured and stored by the patch. (2) Patch data are transferred by Bluetooth to an app on the patient’s paired mobile device. (3) Patients can follow their own medication taking (the DHFS has the capacity to send patients tailored automated reminder messages). (4) Data are transferred to secure servers. (5) Patient-approved health care workers can remotely receive real-time treatment adherence data and follow large cohorts of patients using the secure web-based dashboard.

The first ingestible sensor (IS) technology to capture oral medication adherence was approved by the Food and Drug Administration (FDA) in 2015 [1], followed by the approval of a second sensor variety in 2019 [2]. The major advancement associated with these medical devices is their capture of remote real-time data on actual drug ingestion, in some cases with simultaneous physiological data [3]. These novel digital technologies, in addition to accurate oral dose ingestion confirmation, may also allow bidirectional treatment adherence support [4-6]. The FDA-approved Digital Health Feedback System (DHFS) consists of an IS, external wearable patch, and paired mobile device [1]. It detects and records the timing of ingestion events and physiologic measures [3], which are then automatically uploaded to a secure internet server, allowing patients and health care providers to follow medication taking in real time and facilitate patient-provider communication (see Figure 1) [1,3-6].

IS-enabled tenofovir disoproxil fumarate plus emtricitabine (IS-Truvada) with the DHFS has recently been deployed to capture medication adherence behavior in persons starting oral pre-exposure prophylaxis (PrEP) [7]. This study confirmed that the DHFS is highly accurate, providing valid measures of ingestion with 99.3% (95% CI 97.2%-100%) reliability versus direct observation, similar to findings in a population of patients with tuberculosis (TB) [6,7]. Clinical trials evaluating DHFS use in TB and hepatitis C treatments have demonstrated persistence use, efficacy, and even superiority versus direct observation [5,6]. Adverse events were few and mild, involving skin reactions to the patch, similar to reports from studies in chronic cardiometabolic disease management [3,8].

In the arena of HIV prevention, both providers and patients have described concerns with oral PrEP medication adherence as a barrier to successful implementation [9,10]. However, there are still no highly accurate real-time adherence measurement tools used in clinical practice to guide patient and physician discussions around these concerns. Providers continue to depend principally on self-reported adherence, which is subject to recall bias [11-13] and patient’s efforts to avoid potential negative interactions with their physicians when disclosing nonadherence [14,15], or pharmacy refills, which indicate what a patient has on hand but provide no information on if and when medications are taken [16]. Directly observed therapy, which is primarily used in TB treatment, where a person is observed taking their medication, is highly reliable but personnel and resource intensive, time-consuming, costly [6,17,18], and impractical in chronic management for HIV prevention.

Multiple earlier technologies that supply data on oral medication adherence are available. Medication event monitoring system devices use openings of electronic containers and lids but have well-documented inaccuracies based on mismatches between openings and actual pill taking [19-24]. Smartphone apps incorporating SMS text messaging are based on self-report [25] or send videos [26] for later viewing and assessment. In contrast to these surrogate technologies, ISs signal when medication reaches the gastric track and are able to capture individual daily behavior patterns in real time, providing insight into variations in daily medication adherence. This capacity even has advantages over cumulative metabolite-based adherence measures developed and evaluated in the HIV prevention arena, such as dried blood spots and hair analysis [27,28], which do not allow real-time intervention or capture pattern variations in medication ingestion over time [7]. Such patterns are of importance based on the postdose durations of the therapeutic drug [29,30], which alters the risk of acquiring HIV infection, for example, a week where PrEP is taken once, followed by a week where it is taken daily [31-33].

Regardless of the superior capacities of the DHFS, the success or failure of any digital health innovation often depends on how it is received by the user [34]. Limited medical research exists on the user experience (UX) of persons using IS-based digital technology. UX is considered crucial to product design [34]. Classical consumer research ranks products according to levels of utility to consumers, which are subjective individual tastes; however, individuals change over time, and effort is devoted to developing an understanding of current and future user “personas” [35]. In contrast, traditional medical research, particularly in the infectious disease arena, historically looks for programmatic implementation of adherence technology, with the implicit assumption that “one size” should or could “fit all.” The development of technology acceptance models has underscored the importance of understanding how personal attitudes contribute to behavioral intention on technology use [36-41], particularly how perceived ease of use and utility influences individuals’ willingness to adopt and continue to use a given technology [39]. Research on the contribution of personal attitudes and characteristics is now expanding into health care technology use [42] but is entirely novel in the arena of digital adherence technology.

We conducted a detailed UX evaluation among persons prescribed PrEP to prevent HIV infection who used the DHFS with IS-Truvada. Our study evaluated the overall experience, ease of use, perceived utility, and intention of future use. We captured baseline demographics and urine toxicology screening (UTOX). In addition, we collected detailed self-report questionnaires to evaluate depressive symptoms, HIV risk perception, sleep, and individual self-efficacy in the context of medication taking [43]. Self-efficacy is defined as a person’s belief in their capability to succeed and achieve a given level of performance [44] and is considered to be connected to motivation, achievement, and self-regulation [45,46]. We then conducted an association analysis of our UX findings with individual participant characteristics captured at baseline, to see if current and future user “personas” emerged.

## Methods

### Overview

UX data were collected upon exit from a prospective, single-arm, open-label intervention study of participants using the DHFS (manufacturers: Proteus Digital Health and Otsuka Pharma) with IS-enabled tenofovir disoproxil fumarate plus emtricitabine for up to 12 weeks. The parent intervention study evaluated DHFS adherence measurements, ability to capture patterns of adherence behavior, and the association of predictors with adherence behavior among persons starting PrEP [7].

### Ethical Considerations

The study protocol was approved by the University of California San Diego (UCSD) Institutional Review Board (#161618), was conducted in accordance with Good Clinical Practice principles, and was registered on ClinicalTrials.gov (NCT03693040). Participants signed an informed consent. All data derived from this study were deidentified. Participants were compensated with a gift card equivalent in value to US $50 on the completion of all activities associated with the baseline and study exit visits, which included survey completion.

### Participants

Eligible participants were HIV and hepatitis B seronegative, aged ≥18 years old, were at risk for HIV, and desired oral PrEP. Participants were recruited from the UCSD AntiViral Research Center, UCSD Owen Clinic, or other primary care clinics in San Diego. Participant procedures were as follows. Baseline laboratory evaluations were required within the defined parameters; participants needed to be able to use mobile devices (these were provided by the study if they did not have them), be willing to use the DHFS, and have no known skin adhesive hypersensitivity. Baseline demographics, UTOX, and self-reported questionnaires were collected. Participants were instructed on DHFS use at baseline; this instruction comprised how to place and change the patch, how to pair the patch with the mobile device, and how to connect the mobile device to Wi-Fi. During the trial, participants changed the monitor patch themselves as needed and could view the medication ingestion log on their mobile device. Study staff counseled participants on wearing the patch and keeping their paired mobile device consistently charged. After the intervention, participants underwent repeat HIV testing and continued on PrEP as prescribed by their practitioner. Participants with ≥28 days in study (DHFS with IS-Truvada) completed the detailed exit questionnaire and formed the cohort analyzed.

### Measures

Baseline self-report questionnaires evaluated habitual self-control [47], self-efficacy beliefs [43], depression (Patient Health Questionnaire–8 [PHQ-8]) [48], sleep (Pittsburgh Sleep Quality Index [PSQI]) [49], and HIV risk perception (Perceived Risk of HIV Scale [PRHS] 8-item) [50]. On study exit, the detailed UX questionnaire was completed. The UX questionnaire consisted of 27 questions with responses coded from 1 to 5 and included reverse scored and related questions to ensure validity [51]. Of the items scored on the Likert scale, 2 questions assessed satisfaction, from 1=extremely unsatisfied to 5=extremely satisfied; 5 questions asked participants to rate various aspects of the system, from 1=extremely unhelpful to 5=extremely helpful; and the responses to the remaining questions ranged from 1=strongly disagree to 5=strongly agree. The UX questions were grouped into 4 domain categories: overall experience, ease of use, intention of future use, and perceived utility. Textbox 1 shows the questions, domains, and the number of questions per domain.

Textbox 1. **User experience questionnaire items for the Digital Health Feedback System (DHFS). Domain categories are shown, with questions grouped by category, not in the originally administered order.**

**Overall experience**

1. How would you rate your overall experience with participating in this mediation adherence study?

2. How would you rate your overall satisfaction with the DHFS (the iPad and patch system)?

3. Overall, this experience using the DHFS was positive.

4. Overall, this experience using the DHFS was challenging. (reverse scored)

**Ease of use**

5. When you started the study, how helpful was the Patient Information Booklet?

6. How helpful were the Proteus app instructions?

7. I was very comfortable changing the patch on my own.

8. The instructions for changing the patch were easy to follow.

9. The patch is comfortable to wear.

10. Wearing the patch interfered with my daily activities. (reverse scored)

11. The Proteus app was difficult to navigate. (reverse scored)

12. Accessing my medication ingestion report was difficult. (reverse scored)

13. Technical difficulties were easily resolved.

**Intention of future use**

14. I would use the DHFS in the future.

15. I would use the DHFS in the future to keep track of my treatment.

16. I would use the DHFS in the future if I had problems following my treatment.

17. I would recommend that others use the DHFS.

18. I would recommend use of the DHFS to others if they are having problems following their treatment.

**Perceived utility**

19. How helpful was participating in the study for your medication adherence?

20. How helpful was the DHFS in helping you follow your medication adherence?

21. I used the DHFS app frequently to follow my medication taking.

22. I used the DHFS app frequently to follow my activity and rest.

23. The DHFS was useful.

24. The DHFS made taking my medication easier for me.

25. Using the DHFS interfered with how I typically manage my medications. (reverse scored)

26. Referring to the Proteus app during the study helped me track my medication adherence.

27. The DHFS improved my medication adherence.

### Statistical Analysis

A total of 27 UX questionnaire item responses were scored (using reverse scoring where necessary), so that higher scores mean higher levels of satisfaction. The questions’ average values were used for the total summary score and each of the 4 domain summary subscores. Demographic characteristics of participants who completed the UX questionnaire were compared to those of nonresponders using the Wilcoxon rank-sum test and Fisher exact test as appropriate.

Single- and multipredictor linear models were used to analyze the 5 summary scores for their association with demographic and other baseline characteristics, including age, gender, race and ethnicity (non-Hispanic White, Asian, Black, or Hispanic), UTOX results (positive or negative), number of substances detected in UTOX, sleep (PSQI), self-efficacy, habitual self-control, HIV risk perception (PRHS 8-item), and depression (PHQ-8). Prior to regression analyses, the multiple imputation by chained equations method with 10 imputations was used to impute missing values in the predictors. All model estimates were based on pooled results, using the rules from Rubin [52]. Predictors with *P* values <.20 in the univariable analyses were included for consideration into multivariable analyses. Backward model selection with a .20 threshold for predictor significance was used to select the final multivariable models. The *Result*s section reports model coefficients, their 95% CIs, and the relevant *P* values. The CIs for effects of ethnicity were Bonferroni-adjusted for multiple comparisons. PHQ-8 exhibited nonlinear association with the summary total score and subscores. Natural cubic splines, with the number of knots determined by minimizing the Akaike information criterion, were used to model these associations. Analyses and figures were done using R (version 4.0.3; R Foundation for Statistical Computing) [53]. The R package *mice* was used for multiple imputation analyses [54].

## Results

### Study Cohort Description

#### Study Enrollment, Demographics, and Cohort Description

Overall, 71 persons were enrolled in the intervention using the DHFS with IS-Truvada. Participants had a mean age of 37.6 (range 18-69) years and were mostly male (n=64, 90%), White (n=55, 77%; n=24, 34% were Hispanic), housed (n=68, 96%), and employed (n=53, 75%). Baseline toxicology was positive in 41% (n=28) of participants, with marijuana (n=17, 25%), amphetamines (n=10, 14%), and methamphetamines (n=8, 12%). A total of 63 participants used the DHFS with IS-Truvada for ≥28 days, and there were no significant differences in baseline demographics compared to enrolled participants who dropped out early (n=8) [7]. Of the 63 participants, 53 (84%) fully or partially completed the comprehensive UX exit questionnaire. Table 1 shows the demographic characteristics of participants at baseline and includes the comparison of participants who completed the UX questionnaire and those that did not. Questionnaire respondents did not differ statistically from nonrespondents on age (37.5 vs 33.9 y; *P*=.34), sex (48/53, 91% vs 9/10, 90% male; *P*>.99), employment status (39/53, 74% vs 7/10, 70% employed; *P*>.99), or race and ethnicity (30/53, 57% vs 5/10, 50% non-Hispanic White; *P*=.43). However, questionnaire respondents were more likely to have stable housing (52/53, 98% vs 8/10, 80%; *P*=.06) and less likely to test positive on UTOX (18/51, 35% vs 7/10, 70%; *P*=.08), particularly for methamphetamine (4/51, 8% vs 4/10, 40%; *P*=.02). No significant difference was observed in self-report questionnaire scores between respondents and nonrespondents (all *P*>.05).

**Table 1.** Baseline cohort characteristics and comparison between completers and noncompleters of the user experience questionnaire. *P* values are based on Wilcoxon rank-sum test (numeric variables) and Fisher exact test (categorical variables).

Variable		Completed exit survey (n=53)	Did not complete exit survey (n=10)	*P* value
Age (years), mean (SD)		37.5 (10.8)	33.9 (11.2)	.34
**Gender, n (%)**				>.99
	Male	48 (91)	9 (90)	
	Female or transgender	5 (9)	1 (10)	
**Race and ethnicity, n (%)**				.43
	Asian, non-Hispanic	4 (8)	0 (0)	
	Black, non-Hispanic	3 (6)	2 (20)	
	Hispanic	18 (30)	3 (30)	
	White, non-Hispanic	30 (57)	5 (50)	
Positive drug screen (any drugs), n (%)		18 (35)^a^	7 (70)	.08
Number of drugs identified on toxicology screen, median (IQR)		0.00 (0.00-1.00)^a^	1.00 (0.25-2.75)	.02^b^
Positive methamphetamine toxicology screen, n (%)		4 (8)^a^	4 (40)	.02^b^
**Employment, n (%)**				>.99
	Full or part time	39 (74)	7 (70)	
	Unemployed, retired, or disabled	14 (26)	3 (30)	
**Housing, n (%)**				.06
	Stable	52 (98)	8 (80)	
	Transient or homeless	1 (2)	2 (20)	
Global PSQI^c^ score, mean (SD)		6.35 (3.17)^d^	5.29 (3.55)^e^	.44
Self-efficacy, mean (SD)		4.47 (0.41)^f^	4.42 (0.43)^g^	.67
Habitual self-control, mean (SD)		3.68 (0.60)^h^	3.83 (0.29)^g^	.61
HIV risk perception (PRHS^i^ 8-item), mean (SD)		23.1 (5.49)^d^	24 (8.67)^g^	.50
PHQ-8^j^ total (8-item), median (IQR)		3.0 (1.0-5.0)^k^	1.0 (0.0-2.0)^l^	.07

a n=51.

b *P*<.05.

c PSQI: Pittsburgh Sleep Quality Index (higher score=worse).

d n=49.

e n=7.

f n=45.

g n=8.

h n=43.

i PRHS: Perceived Risk of HIV Scale (higher score=worse).

j PHQ-8: Patient Health Questionnaire–8 (higher score=worse).

k n=50.

l n=9.

#### UX Questionnaire Summary Scores

Table 2 lists the summary statistics, including mean and range, for the total summary score and the 4 themed subscores. On average, participants expressed an overall satisfaction with the DHFS system (total summary score: mean 3.74, SD 0.70). On average, the participants had the highest summary score for overall experience (mean 3.89, SD 0.87), followed by ease of use (mean 3.74, SD 0.65), perceived utility (mean 3.73, SD 0.76), and intention of future use (mean 3.58, SD 1.08). Based on IQR values, at least 75% of participants provided favorable feedback for the total score (IQR 3.17-4.20), overall experience (IQR 3.50-4.50), ease of use (IQR 3.33-4.22), and perceived utility (IQR 3.22-4.25), and at least 50% of participants expressed favorable feedback on intention of future use (IQR 2.80-4.40).

**Table 2.** Summary scores from the user experience questionnaire.

Summary score	Participants, n	Value, mean (SD)	Value, median (IQR)	Value, range
Overall experience	53	3.89 (0.87)	4.00 (3.50-4.50)	2.25-5.00
Ease of use	52	3.74 (0.65)	3.72 (3.33-4.22)	2.33-5.00
Intention of future use	53	3.58 (1.08)	3.80 (2.80-4.40)	1.00-5.00
Perceived utility	48	3.73 (0.76)	3.72 (3.22-4.25)	1.44-5.00
Total	47	3.74 (0.70)	3.78 (3.17-4.20)	2.00-4.96

### Participant Characteristics as Predictors of UX Score

#### Total Summary Score

The single-predictor analyses showed that higher total summary scores were associated with better self-efficacy rating (0.822 per point, 95% CI 0.405-1.240; *P*<.001) and PHQ-8 score with a nonlinear trend (natural cubic spline with 1 knot). Only self-efficacy remained in the model after multipredictor model selection. Regression analyses for predictors of total summary score are shown in Table 3.

**Table 3.** Single- and multipredictor linear regression associations of demographics and baseline characteristics with the total user experience score.

Variable		Single-predictor model		Multipredictor model	
		Coefficient (95% CI)	*P* value	Coefficient (95% CI)	*P* value
Age (per year)		0.008 (−0.011 to 0.027)	.38	—^a^	—
**Gender**			.60		—
	Male	Reference		—	
	Female or transgender	−0.197 (−0.944 to 0.549)		—	
**Race and ethnicity**			.71		—
	Asian, non-Hispanic	−0.122 (−0.944 to 0.701)		—	
	Black, non-Hispanic	0.320 (−0.705 to 1.344)		—	
	Hispanic	0.209 (−0.377 to 0.796)		—	
	White, non-Hispanic	Reference		—	
Positive drug screen (any drugs)		0.214 (−0.239 to 0.666)	.35	—	—
Number of drugs (per drug)		0.103 (−0.139 to 0.346)	.39	—	—
Global PSQI^b^ score (per point)		−0.018 (−0.082 to 0.047)	.58	—	—
Self-efficacy (per point)		0.822 (0.405 to 1.240)	<.001	0.822 (0.405 to 1.240)	<.001
Habitual self-control (per point)		0.224 (−0.134 to 0.581)	.21	—	—
HIV risk perception (PRHS^c^ 8-item; per point)		−0.015 (−0.055 to 0.025)	.45	—	—
PHQ-8^d^ total (per point)		—^e^	.16	—	—

a Not applicable.

b PSQI: Pittsburgh Sleep Quality Index.

c PRHS: Perceived Risk of HIV Scale.

d PHQ-8: Patient Health Questionnaire–8.

e Nonlinear terms using cubic spline curves.

#### Overall Experience Score

In the univariable analyses, associations were found at the .20 significance level between a higher overall experience score and male gender (mean difference 0.543, 95% CI −0.274 to 1.360; *P*=.19 vs female or transgender), Hispanic ethnicity (mean difference 0.654, 95% CI 0.026-1.282 vs non-Hispanic White), better PSQI score (−0.080 per point, 95% CI −0.152 to −0.007; *P*=.03), better self-efficacy rating (0.688 per point, 95% CI 0.146-1.230; *P*=.01), stronger habitual self-control (0.384 per point, 95% CI −0.008 to 0.776; *P*=.06), and PHQ-8 score with a nonlinear trend (natural cubic spline with 1 knot; *P*=.09). In the multivariable analyses, incorporating the above univariable associations of .20 significance and higher, the overall experience score was associated with race and ethnicity (*P*=.02), lower PSQI score indicating better sleep (−0.070 per point, 95% CI −0.133 to −0.006; *P*=.03), and higher self-efficacy rating (0.771 per point, 95% CI 0.292-1.250; *P*=.002; see Table 4).

**Table 4.** Multipredictor linear regression associations of demographic, baseline characteristics, and percentage of confirmed doses with subscores. See Figure 2 for the perceived utility model.

Variable		Overall experience		Ease of use		Intent of future use		Perceived utility	
		Coefficient (95% CI)	*P* value	Coefficient (95% CI)	*P v*alue	Coefficient (95% CI)	*P* value	Coefficient (95% CI)	*P* value
**Race and ethnicity**			.02		—^a^		—		—
	Asian, non-Hispanic	−0.195 (−1.094 to 0.704)		—		—		—	
	Black, non-Hispanic	0.937 (−0.209 to 2.083)		—		—		—	
	Hispanic	0.606 (0.002 to 1.210)		—		—		—	
	White, non-Hispanic	Reference		—		—		—	
Global PSQI^b^ score (per point)		−0.070 (−0.133 to −0.006)	.03	—	—	—	—	—	—
Self-efficacy (per point)		0.771 (0.292 to 1.250)	.002	0.750 (0.375 to 1.126)	<.001	0.885 (0.213 to 1.557)	.01	0.901 (0.411 to 1.391)	<.001
PHQ-8^c^ total (per point)		—	—	—	—	—	—	—^d^	.01

a Not applicable.

b PSQI: Pittsburgh Sleep Quality Index.

c PHQ-8: Patient Health Questionnaire–8.

d Nonlinear terms using cubic spline curves.

**Figure 2.**
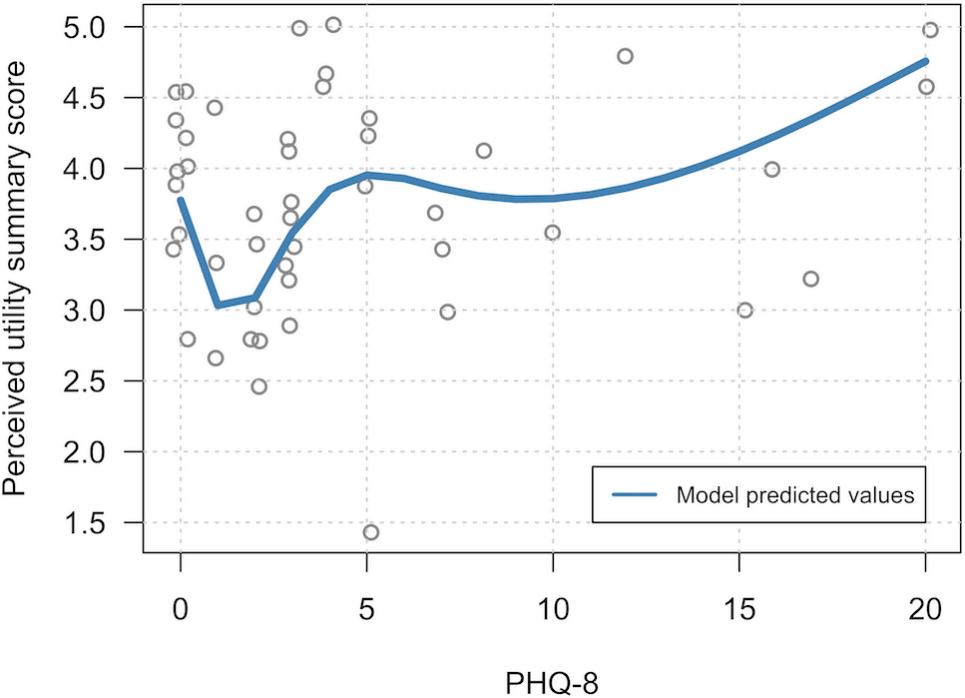
Observed values (points) and the predicted spline (line) showing association between PHQ-8 and perceived utility summary score, adjusted for self-efficacy. PHQ-8: Patient Health Questionnaire–8.

#### Ease of Use Score

The ease of use score showed correlation with better self-efficacy rating (0.750 per point, 95% CI 0.375-1.126; *P*<.001) and PHQ-8 score with a nonlinear trend (natural cubic spline with 1 knot; *P*=.16). The multipredictor model on the ease of use score retained only self-efficacy as a predictor (see Table 4).

#### Intention of Future Use Score

Only the self-efficacy rating was associated with the intention of future use score (0.885 per point, 95% CI 0.213-1.557; *P*=.01; see Table 4)

#### Perceived Utility Score

The single-predictor analyses showed association between the perceived utility score and self-efficacy rating (0.672 per point, 95% CI 0.187-1.157; *P*=.008), as well as PHQ-8 score (natural cubic spline with 3 knots; *P*=.10). The multipredictor model retained both predictors and showed that better perceived utility score was associated with better self-efficacy rating (0.901 per point, 95% CI 0.411-1.391; *P*<.001) and worse PHQ-8 score (natural cubic spline with 3 knots; overall *P*=.01; see Table 4).

## Discussion

### Principal Findings

The detailed UX analysis indicated that greater than 75% of participants who used the DHFS with IS-Truvada reported positive experiences, based on the total score and the subscore analyses (overall experience, ease of use, and perceived utility analyses); 70% of participants responded positively toward the intention of future use. Multivariable linear regression analyses of participant characteristics found that having a higher baseline self-efficacy concept score was associated with more positive UX in the total score and across all subscores. In addition, Hispanic ethnicity and more depressive symptoms were associated with reporting positive overall experience and higher DHFS utility, respectively. Poor sleep (captured by the global PSQI score) was associated with a worse overall experience.

UX research is central to the process of developing user-centric technology integration into clinical arenas serving different patient populations [34]. Our research indicates that among participants prescribed PrEP, the DHFS with IS-Truvada was well received. Meta-analysis of UX with mobile health technology repeatedly finds the following themes as being critical to the end user: functionality (related to experiences supporting self-management); acceptance (related to usability and feasibility); perceptions of benefit (related to self-efficacy and empowerment); and importance of co-design [55]. From this perspective, our findings indicate that the DHFS met critical end-user needs of functionality, acceptance, and perception of benefit in the population using PrEP in this study.

Our data currently represent one of the largest and most detailed study available on UX in persons with any medical diagnosis who have experience using an IS medication adherence system. Our findings are in line with those of Chai et al [56], who reported from qualitative interviews that 15 out of 90 persons using a digital pill system (DPS) [2] for PrEP adherence measurement perceived the device as acceptable, novel, and valuable. Interestingly, men who have sex with men (MSM) taking PrEP who reported substance use were also found to have positive perceptions toward using the DPS in the future [57]. Notably, the PrEP cohort we studied had significantly higher levels of UX satisfaction with the DHFS than that reported in participants in a psychiatric study population (greater than 75% vs 53%, respectively) [58]. The authors noted that their study population included participants with acute psychotic illness; in contrast, participants on stable antipsychotic doses without psychotic symptoms in a prior study [59] reported 70% satisfaction and 78% utility in response to single-question item. The findings from a stable psychiatric study population are close to those found by our detailed UX analysis in persons starting PrEP.

At the outset, we investigated whether baseline demographics and self-report questionnaires could be used to inform current and future use “personas” among our study population, following the expectation that with technology use, “one size may not fit all.” A significant association with positive UX for the total score and all subscores was the participants’ sense of self-efficacy. Self-efficacy is defined as a person’s belief in their capability to succeed and achieve a given level of performance [44]. Self-efficacy is considered to be connected to motivation, achievement, and self-regulation [45,46]. We used an established scale for capturing self-efficacy in the context of medication taking [43] and found an association between the self-efficacy concept and experience of DHFS technology functionality, acceptance, and perception of benefit. Our findings indicate that the self-efficacy concept is directly related to the use of the health care technology tested, and our findings are in line with prior reports showing that self-efficacy beliefs can affect perceived usefulness and perceived ease of use of technology in general, and health informatics and digital health social media applications in particular [60,61].

Persons having lower sleep quality at baseline reported a worse overall experience with the DHFS, which may be related to the requirement of a patch worn on the torso in the system tested and suggests that sleep quality should be evaluated before using the DHFS in a clinical study or practice. The role of depressive symptoms on the UX with the DHFS needs more evaluation. Current evidence indicates that persons with depressive symptoms have significantly higher use of health information technology than persons with other chronic diseases [62], and a plethora of research exists on both digital data-gathering and web-based intervention tools for depression [63]. No published literature appears to be available on what persons with depressive symptoms or a diagnosis of depression *want* from digital health technology. It is likely such data are collected during technology “co-design” efforts, but these data may be analyzed as chronic disease comorbidities or general mental health associations. Our findings suggest that specific criteria for digital health technology may be important to persons with depressive symptoms and support separate analysis of user requirements and experience for these persons.

### Clinical Implications

As with any long-term therapy, successful provision of PrEP to prevent HIV infection requires a compassionate mindset, involving a highly individualized series of investigations into how each patient and their disease risk intertwine over time, with trust and honesty between both patients and physicians [64]. Patients need and have choices on PrEP delivery. Based on our UX evaluation of the DHFS with IS-Truvada, this technology is acceptable to patients prescribed PrEP and may be useful to provide insights for both patients and providers on optimal PrEP treatment modalities for individuals over time [7].

### Limitations

The study sample was almost exclusively comprised of MSM, and the findings are not generalizable to other populations of patients using PrEP. The study duration was limited to 3 months, and our analysis included only those who persisted with the technology for at least 1 month, 84% (53/63) of whom completed the detailed UX questionnaire. As is expected with a detailed questionnaire, some participants omitted answers to some questions. However, the use of reverse scored and related questions, while primarily designed to ensure questionnaire validity, also served to expand the capture of concepts where questions were left unanswered. Significant differences were observed between questionnaire nonrespondents and respondents, limiting the generalizability of our findings to persons taking PrEP who are homeless or use methamphetamine. In the latter regard, it is notable that Chai et al [56,57] report positive UX and attitude findings regarding DPS technology in MSM taking PrEP who use substances. Despite being one of the largest detailed study available on UX in persons who used an IS medication adherence system, our sample size was limited, and as the probability of reporting a positive experience was observed in >75% of the study population, the likelihood of identifying “current and future use personas” across our study was restricted by our sample size.

### Conclusions

The evaluated IS technology met critical end-user needs of functionality, acceptance, and perception of benefit in the population using PrEP in this study. These findings support the continued evaluation of IS adherence technologies in this patient population. Oral medication adherence is a complex behavior. Increased focus on understanding and matching the needs of individual patients to available digital adherence technology options may improve the impact of these technologies on adherence monitoring and support, as well as inform optimal PrEP treatment modalities for individual patients over time.

## Abbreviations

DHFS: Digital Health Feedback System
DPS: digital pill system
FDA: Food and Drug Administration
IS: ingestible sensor
MSM: men who have sex with men
PHQ-8: Patient Health Questionnaire–8
PrEP: pre-exposure prophylaxis
PRHS: Perceived Risk of HIV Scale
PSQI: Pittsburgh Sleep Quality Index
TB: tuberculosis
UCSD: University of California San Diego
UTOX: urine toxicology screening
UX: user experience
